# Postoperative pneumocephalus as a predictor of chronic subdural hematoma recurrence: a retrospective cohort analysis

**DOI:** 10.1007/s00068-025-02939-y

**Published:** 2025-07-21

**Authors:** Haitham Alenezi, Tim Lampmann, Harun Asoglu, Mohammed Jaber, Mohammed Banat, Hartmut Vatter, Lars Eichhorn, Motaz Hamed

**Affiliations:** 1 Department of Neurosurgery, University Hospital Bonn, Bonn, Germany; 2 Department of Anesthesiology, Intensive Care Medicine and Pain Medicine, Helios Hospital Bonn/Rhein-Sieg, Bonn, Germany

**Keywords:** Chronic subdural hematoma, Recurrence, Outcome, Pneumocephalus

## Abstract

**Background:**

Chronic subdural hematoma (CSDH) is a common neurosurgical condition, especially in the elderly, which is usually diagnosed by computed tomography (CT) scan and often treated surgically. After surgery patients shows favourable outcomes with improvement in symptomatology. Despite the availability of various surgical techniques, complications continue to occur frequently due to the advanced age of patients and underlying medical conditions. Burr-hole craniotomy followed by placement a closed-system drainage is widely considered the best treatment for CSDH, although technical challenges and a high recurrence rate remain. Postoperative pneumocephalus is a potential risk factor for recurrence. While the majority of patients recover well after surgery, recurrence or persistence of CSDH occurs in 0.35–33% of cases, sometimes necessitating additional surgeries.

**Method:**

This retrospective study comprehensively evaluates the clinical data of 229 patients diagnosed with chronic subdural hematoma (CSDH) who underwent surgical intervention, specifically burr-hole craniotomy with drainage between 2016 and 2021. The primary objective is to measure the prognostic significance of postoperative pneumocephalus as a predictor of recurrence of CSDH. Furthermore, the obtained univariate and multivariate regression analyses examines various patient-specific factors, including age, gender, location of CSDH (unilateral or bilateral), anticoagulation therapy status, neurosurgical follow-up outcomes, hospital readmission rates, and the incidence of repeat surgical procedures.

**Result:**

Among the analysed characteristics, postoperative pneumocephalus exceeding a specified volumetric threshold emerges as the only significant predictor of CSDH recurrence. This recurrence of CSDH is additionally associated with a substantial prolongation of the patient’s hospitalization, highlighting its clinical and logistical significance.

**Conclusion:**

A postoperative pneumocephalus is nearly unavoidable; however, optimizing surgical technique to minimize its volume below 5.2 cm^3^ reduces significantly the recurrence rate of CSDH.

## Introduction

Chronic subdural hematoma (CSDH) is a common and clinically significant neurosurgical condition, particularly affecting the elderly population [1–4]. It is characterised by the accumulation of blood in the subdural space, often resulting from minor head trauma, anticoagulation therapy, or age-related brain atrophy [1–8]. Diagnosis is typically based on cranial computed tomography (CCT) scans, which provide clear imaging of the hematoma and allow adequate assessment of treatment [8–10]. Based on clinical and radiological findings is the selected treatment a surgical intervention, which generally leads to favourable outcomes, including symptom resolution and restoration of functional independence in the majority of cases [6, 8, 10, 11]. Despite the availability of various surgical techniques, complications are frequent due to patients’ advanced age and medical conditions. Among the various techniques, burr-hole craniotomy followed by placement of a closed-system drainage is widely regarded as the gold standard for CSDH treatment due to its balance of efficacy, safety, and simplicity [3, 7, 10, 11]. However, even with this approach, challenges persist. Technical difficulties during the procedure and a recurrence rate ranging from 0.35 to 33% appears as a barrier in achieving optimal patient outcomes [2, 8, 12, 13]. Postoperative pneumocephalus, defined as the presence of air in the cranial cavity after surgery, has been identified as a potential risk factor for recurrence of CSDH [3, 4, 8, 10]. This condition may interfere with the CSDH and increase the probability of reaccumulation, necessitating further surgical interventions [2, 5, 8, 11, 13]. While the majority of patients recover well following their initial surgery, the recurrence or persistence of CSDH remains a substantial concern, which frequently require a repeat procedures [9, 14, 15]. This emphasise the importance of identifying possible modifiable risk factors, optimizing surgical techniques, and enhancing postoperative care to minimize complications and improve long-term outcomes.

## Materials and methods

### Study design

Between 2016 and 2021, a total of 229 patients with CSDH underwent burr-hole craniotomy at our centre. The indication of surgery made based on clinical symptoms and radiological findings. In the included cases exceeds the maximum width of the CSDH the adjacent skull width by at least one hematoma width. Baseline characteristics, including patient age, sex, anticoagulant use, volume of postoperative pneumocephalus, recurrence rates, and the incidence of reoperation, were retrospectively analysed. Given the substantial variability observed in postoperative pneumocephalus data, the focus of this study is on the postoperative findings. Specifically, it aims to determine the threshold at which postoperative pneumocephalus becomes clinically relevant in predicting recurrence and to identify reliable methods for its accurate measurement. Additionally, the study aims to explore whether other factors contribute to the development of postoperative pneumocephalus, thereby providing a widely understanding of its clinical implications.

### Clinical management

Following neurosurgical evaluation based on clinical presentation and imaging findings, patients underwent one burr-hole craniotomy to evacuate the CSDH. The intraoperative decision to place one or more drainage systems (Blake drain) was made according to the specific surgical and anatomical requirements identified during the procedure. Postoperative a CCT scan was performed within 72 h after surgery, guided by the patient’s clinical condition, and was typically followed by the removal of the drainage system after imaging confirmation.

The volume of postoperative pneumocephalus was quantified using measurements derived from axial, coronal, and sagittal planes of the CCT by applying the formula (maximum length x maximum width x maximum height). For following clinical and morphological assessment, patients underwent a follow-up CCT scan approximately 3 to 6 weeks post-surgery. In cases where symptomatic abnormalities were suspected, the follow-up CCT was expedited. If symptomatic recurrence of CSDH was identified during follow-up imaging, reoperation was promptly performed to resolve the condition.

### Statistical analysis

Continuous data were expressed as mean ± standard deviation and categorical variables were expressed as absolute and relative frequencies. Unpaired two-tailed t-test was used for differences of continuous data whereas categorical variables were analysed in contingency tables using the Fisher exact test. Receiver-operating characteristic (ROC) analysis was performed to evaluate how postoperative pneumocephalus was associated with recurrence rate of CSDH. Youden’s J statistic was used to select the best cut-off point. P-values < 0.05 were considered statistically significant. All calculations were performed by SPSS Statistics (Version 29, IBM Corp. Armonk, NY, USA). The software iPlan (Brainlab Inc., Felkirchen, Germany) was used for the measurement of postoperative pneumocephalus in the postoperative CCT-scans.

## Results

All 229 patients with chronic subdural hematoma (CSDH) who underwent a burr-hole craniotomy at our centre exhibited a specific volume of postoperative pneumocephalus. The mean age at diagnosis was 78.2 years, with 32.3% of patients being female and 67.7% male. Among these cases, 27.9% presented with bilateral CSDH, while 38.9% had right-sided and 38.9% had left-sided CSDH. A history of platelet aggregation inhibitor use was noted in 39.7% of patients, and 27.5% had a history of direct oral anticoagulant use. CSDH recurrence occurred in 20.5% of patients within 3 to 6 weeks postoperatively, necessitating reoperation in 93.6%, due to the symptomatic relevant recurrent CSDH in these cases. Additionally, 4.8% of patients died during their hospitalization (Table 1).

**Table 1:** Patient characteristics

	*N* (%)
Overall	229 (100%)
Mean Age at Diagnosis in years (IQR)	78.2 (76.9–79.5)
Sex	
Female	74 (32.3%)
Male	155 (67.7%)
Side	
Right	76 (33.2%)
Left	89 (38.9%)
Bilateral	64 (27.9%)
Placement of Subdural Drainage	
No subdural drainage	16 (6.9%)
1x subdural drainage 2x subdural drainage	112 (48.9%) 101 (44.2%)
Platelet Aggregation Inhibitor Yes No	91 (39.7%) 138 (60.3%)
Direct Oral Anticoagulants (DOACs)	
Yes	63 (27.5%)
No	166 (72.5%)
Postoperative Recurrence	47 (20.5%)
Reoperation	44 (93.6%)
Mortality during Hospitalization	11 (4.8%)

The table summarise of the clinical characteristics and outcomes of 229 patients diagnosed with CSDH and underwent a burr-hole craniotomy. The variables analysed include patient age at diagnosis, gender, location of the hematoma, use of platelet aggregation inhibitors, use of direct oral anticoagulants, incidence of postoperative recurrence as assessed by clinical evaluation and imaging within 3 to 6 weeks, the number of preoperative interventions, length of hospital stay, and in-hospital mortality rate with standard deviation (SD)

**Fig. 1:**
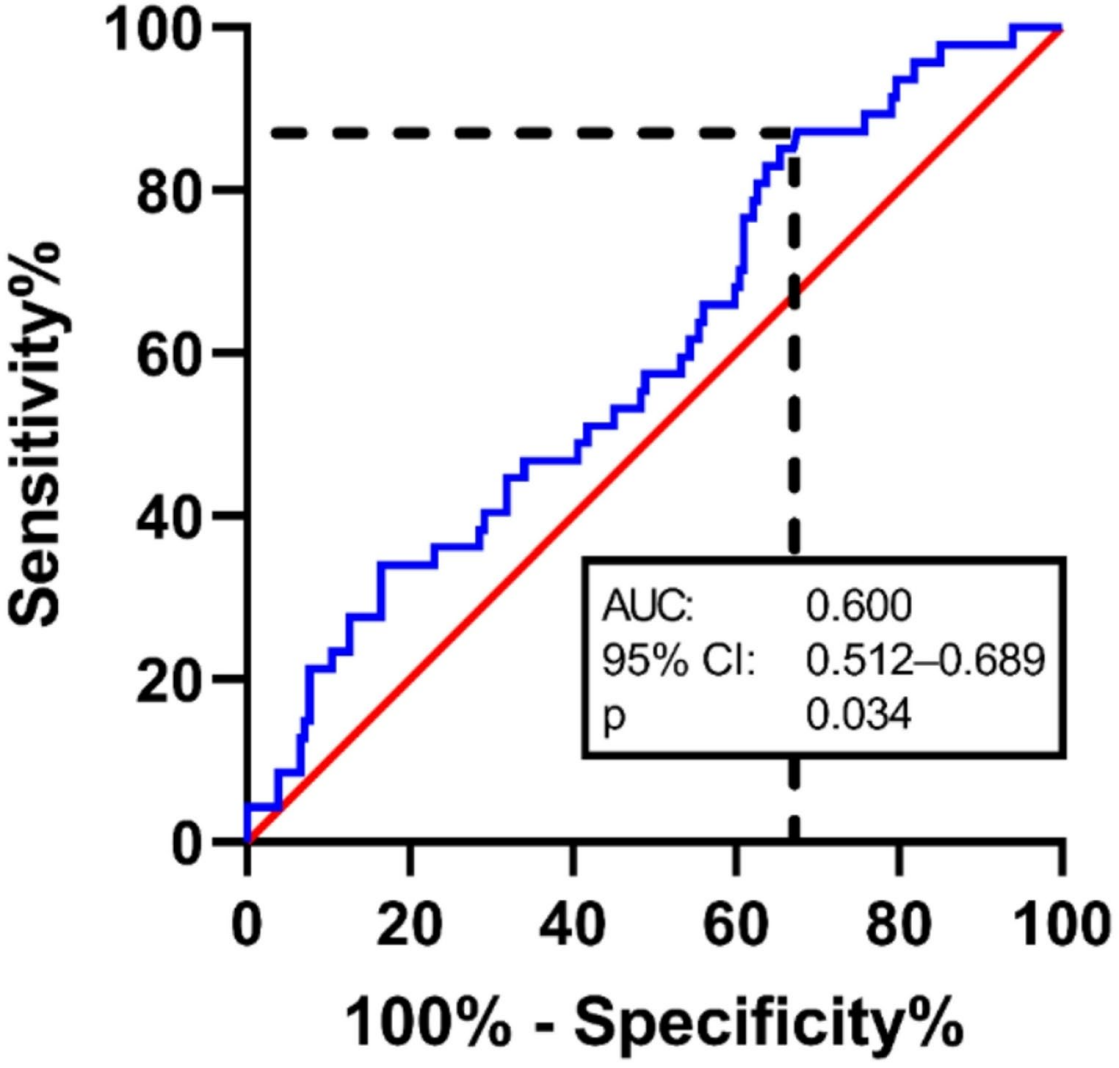
ROC-Analysis. The receiver operating characteristic (ROC) curve presented in Fig. 1 visualise the relationship between the volume of postoperative pneumocephalus and the recurrence of chronic subdural hematoma. The analysis performed by Youden’s J statistic identifies a cut-off volume of 5.2 cm³, at which sensitivity and specificity are optimally balanced

The receiver operating characteristic (ROC) curve visualised in Fig. 1, demonstrates the relationship between the volume of postoperative pneumocephalus and the recurrence of chronic subdural hematoma (CSDH). Using Youden’s J statistic, the analysis identifies an optimal cut-off volume of 5.2 cm³, which achieves the best balance between sensitivity and specificity for predicting recurrence (Table 2).

**Table 2:** Comparison between the postoperative recurrence group and the no postoperative recurrence group

	Postoperative Recurrence	No Postoperative Recurrence	*p*-value
Overall	47 (20.5)	182 (79.5)	
Age	75.7 ± 9.3	78.9 ± 10.2	0.054
Sex			0.729
Female	14 (29.8%)	60 (33.0%)	
Male	33 (70.2%)	122 (67.0%)	
Side			0.838
Right	15 (31.9%)	61 (33.5%)	
Left	20 (42.6%)	69 (37.9%)	
Bilateral	12 (25.5%)	52 (28.6%)	
Platelet Aggregation Inhibitor	19 (40.4%)	72 (39.6%)	1.000
Direct Oral Anticoagulants (DOACs)	12 (25.5%)	51 (28.0%)	0.855
Duration of Hospitalisation	11.5 ± 12.8	7.9 ± 5.9	**0.006**
Mortality during Hospitalisation	4 (8.5%)	7 (3.8%)	0.243
Placement of postoperative Drainage	43 (91.5%)	170 (93.4%)	0.748
Volume of postoperative Pneumocephalus			**0.014**
blew 5.2 cm³	8 (17.0%)	65 (35.7%)	
above 5.2 cm³	39 (83.0%)	117 (64.3%)	

The table above presents a comparison between two groups of patients with chronic subdural hematoma (CSDH) who underwent burr-hole craniotomy. The first group had a recurrence, while the second group did not. The comparison evaluates various criteria and characteristics, including age, gender, location of the CSDH, history of anticoagulant or antiplatelet medication use, length of hospital stay, in-hospital mortality, and the volume of postoperative pneumocephalus. In particular, the previously established threshold cut-off volume of postoperative pneumocephalus (5.2 cm³) is compared between both groups

For the comparison, a two-tailed t-test was applied for the comparison between both groups (postoperative Recurrence vs. non-postoperative Recurrence). The volume of postoperative pneumocephalus, appeared companionless as significant difference between the group that experienced recurrence and the group that did not (*p* = 0.014). This difference was remarkably reflected in the length of hospital stay, which also showed a significant variation between the two groups (*p* = 0.006). The other involved criteria Age, Sex, localisation of CSDH (right, left or bilateral), history of Anticoagulation, postoperative drainage showed no significant difference between both groups.

## Discussion

Chronic subdural hematoma is among the most frequently encountered conditions in neurosurgery [2, 3, 5, 14]. It typically appears following head trauma or spontaneously in individuals taking anticoagulant or antiplatelet medications [3, 12, 14]. Common symptoms of chronic subdural hematoma includes among others psychomotor slowing, seizures, personality changes, or focal neurological deficits [1, 6, 8, 9, 16]. Based on clinical presentation and radiological findings, the standard treatment typically involves burr-hole craniotomy for evacuation of the hematoma, followed by the placement of one or two subdural drains to ensure continuous drainage and prevent reaccumulation [10, 11].

A common and challenging complication associated with this procedure is postoperative pneumocephalus [2, 13, 15]. This phenomenon is nearly ensured, as demonstrated in this study, where all 229 patients exhibited postoperative air accumulation within the cranial cavity in the cranial computed tomography (CCT). Furthermore, postoperative pneumocephalus following brain surgery is associated with adverse postoperative outcomes, including an increased incidence of postoperative delirium and a prolonged hospital stay, as demonstrated in this study [2, 6, 15, 17, 18].

While postoperative pneumocephalus after a burr-hole craniotomy is generally asymptomatic and resolves spontaneously in most cases without causing significant clinical issues, its severity can vary [6, 7]. Importantly, the volume of postoperative pneumocephalus has been shown to correlate with worse patient outcomes [4, 18]. Specifically, larger volumes of pneumocephalus are associated with higher recurrence rates of CSDH and a greater need for reoperation [11, 13]. Interestingly, other patient characteristics, including gender, age, laterality of the subdural hematoma (unilateral or bilateral), and anticoagulant usage, did not significantly influence recurrence rates of CSDH in this study.

Postoperative CCT consistently detected pneumocephalus in all cases, prompting the investigation into a threshold volume above which the risk of clinically significant recurrence increases. The study identified a critical threshold of 5.2 cm³, beyond which the probability of recurrence rises significantly. This finding has important clinical implications.

By defining a threshold volume of 5.2 cm³ for postoperative pneumocephalus, clinicians can better predict recurrence risk and apply postoperative management strategies accordingly. Although the complete prevention of postoperative pneumocephalus is impractical, efforts to minimize its volume can result significant benefits. Strategies for volume reduction primarily focus on intraoperative techniques, including thorough irrigation of the surgical site, meticulous closure techniques to limit air entry, and optimal positioning and placement of subdural drains to facilitate effective drainage.

Given the specific characteristics of the study population, primarily elderly patients, in whom multimorbidity and reduced mobility are frequently observed, the postoperative follow-up visits may pose a substantial burden. In light of this, the necessity for additional follow-up in asymptomatic patients with postoperative pneumocephalus measuring less than 5.2 cm^3^ may be limitid. This not only reduces radiation exposure, providing a significant protective benefit, but also confers logistical advantages for both patients and health care provider by eliminating unnecessary monitoring.

In summary, defining such a threshold of postoperative pneumocephalus provides valuable guidance in clinical practice. Although occur of postoperative pneumocephalus is almost unavoidable, its size can significantly impact patient outcomes. Therefore, the focus shifts from preventing pneumocephalus entirely to minimizing its volume as much as possible. This objective can improve outcomes by reducing recurrence rates and the need for reoperation, taking into consideration the importance of precise surgical techniques and vigilant postoperative management.

## Conclusion

This retrospective study of 229 chronic subdural hematoma (CSDH) patients treated with burr-hole craniotomy identifies postoperative pneumocephalus as a critical prognostic factor for CSDH recurrence. The study highlights that postoperative pneumocephalus exceeding a specific volume (> 5.2 cm^3^) is the only significant predictor of CSDH recurrence among the patient-specific factors analysed, including age, gender, CSDH location, and anticoagulation therapy status. Recurrence is associated with prolonged hospitalization, emphasizing its impact on clinical outcomes and healthcare resource. These findings highlight the importance of intraoperative managing postoperative pneumocephalus to minimize recurrence risk and improve patient care efficiency.

## Limitations

This is a retrospective monocentric study. Patients who did not attend follow-up appointments, those who deceased following discharge, and those who declined surgical intervention were excluded from the study.

## Data Availability

No datasets were generated or analysed during the current study.

## Declarations

### Conformations

This manuscript has not been published elsewhere and is not under consideration by another journal.
